# Oxidative and Excitatory Mechanisms of Developmental Neurotoxicity: Transcriptional Profiles for Chlorpyrifos, Diazinon, Dieldrin, and Divalent Nickel in PC12 Cells

**DOI:** 10.1289/ehp.0800251

**Published:** 2008-12-05

**Authors:** Theodore A. Slotkin, Frederic J. Seidler

**Affiliations:** Department of Pharmacology and Cancer Biology, Duke University Medical Center, Durham, North Carolina, USA

**Keywords:** chlorpyrifos, diazinon, dieldrin, excitotoxicity, glutamate receptors, glutathione, metal neurotoxicity, microarrays, nerve growth factor, neuronal development, neurotoxicity, nickel, organochlorine insecticides, oxidative stress, organophosphate insecticides, PC12 cells

## Abstract

**Background:**

Oxidative stress and excitotoxicity underlie the developmental neurotoxicity of numerous chemicals.

**Objectives:**

We compared the effects of organophosphates (chlorpyrifos and diazinon), an organo-chlorine (dieldrin), and a metal [divalent nickel (Ni^2+^)] to determine how these mechanisms contribute to similar or dissimilar neurotoxic outcomes.

**Methods:**

We used PC12 cells as a model of developing neurons and evaluated transcriptional profiles for genes for oxidative stress responses and glutamate receptors.

**Results:**

Chlorpyrifos had a greater effect on oxidative-stress–related genes in differentiating cells compared with the undifferentiated state. Chlorpyrifos and diazinon showed significant concordance in their effects on glutathione-related genes, but they were negatively correlated for effects on catalase and superoxide dismutase isoforms and had no concordance for effects on ionotropic glutamate receptors. Surprisingly, the correlations were stronger between diazinon and dieldrin than between the two organophosphates. The effects of Ni^2+^ were the least similar for genes related to oxidative stress but had significant concordance with dieldrin for effects on glutamate receptors.

**Conclusions:**

Our results point to underlying mechanisms by which different organophosphates produce disparate neurotoxic outcomes despite their shared property as cholinesterase inhibitors. Further, apparently unrelated neurotoxicants may produce similar outcomes because of convergence on oxidative stress and excitotoxicity. The combined use of cell cultures and microarrays points to specific end points that can distinguish similarities and disparities in the effects of diverse developmental neurotoxicants.

A wide variety of toxicants elicit cell damage through their shared ability to produce oxidative stress (Gitto et al. 2002; Gupta 2004; Ohtsuka and Suzuki 2000; Olanow and Arendash 1994). The brain is especially vulnerable because it has a high rate of oxygen consumption, combined with a membrane lipid composition that is enriched in oxidizable polyunsaturated fatty acids (Gupta 2004). The developing brain is even more sensitive because it has lower reserves of protective enzymes and antioxidants (Gupta 2004) and has a higher ratio of neurons to glia, the cells that ordinarily protect neurons from oxidative molecules (Tanaka et al. 1999), while at the same time facing the increased metabolic demand associated with growth. Further, the fact that fetal arterial blood has substantially lower O_2_ concentrations means that the fetal brain is already hypoxic relative to that of a newborn or adult (Faber et al. 1985; Lagercrantz and Slotkin 1986), thus reducing the margin of safety for any agent that compromises oxidative metabolism. The combination of these factors explains why many environmental contaminants elicit oxidative stress within the developing brain (Henderson et al. 1999; Jett and Navoa 2000; Kern and Jones 2006; Mulholland et al. 2005; Pardo and Eberhart 2007; Sinha et al. 2006; Tata and Yamamoto 2007); indeed, this may provide a mechanism by which diverse compounds converge on common sets of neurodevelopmental disorders, such as autism (Kern and Jones 2006; Pardo and Eberhart 2007).

Exposure of the human population to organophosphate pesticides is ubiquitous (Barr et al. 2005; Bouvier et al. 2005; Casida and Quistad 2004), and these compounds are undergoing restriction because of their propensity to produce developmental neurotoxicity [U.S. Environmental Protection Agency (EPA) 2000, 2002, 2006]. Chlorpyrifos, diazinon, and other organophosphates produce oxidative stress in the developing brain, leading to shifts in expression and function of antioxidant genes, and accordingly, antioxidant therapy can offset some of the damage (Bagchi et al. 1995; Giordano et al. 2007; Jett and Navoa 2000; Qiao et al. 2005; Slotkin et al. 2007a; Slotkin and Seidler 2007). Nevertheless, it is increasingly clear that the various organophosphates do not produce the same patterns of neurodevelopmental damage or behavioral deficits, in part because they differ in other mechanisms that contribute to the net adverse outcomes (Pope 1999; Roegge et al. 2008; Slotkin 1999, 2004, 2005; Slotkin et al. 2006a, 2008a, 2008b, 2008c, 2009; Slotkin and Seidler 2007, 2008; Timofeeva et al. 2008a, 2008b). The participation of these additional mechanisms means that, although related compounds may produce similar degrees of oxidative stress, the cellular reactions to that stress may end up being substantially different, such that for the same degree of initial damage, the outcomes may be worse for particular agents. In the present study, we tested that hypothesis by examining the transcriptional responses to chlor pyrifos and diazinon for the various cellular targets involved in the response to oxidative stress: catalase (*cat*), the isoforms of superoxide dismutase (*sod*), glutathione synthase (*gss*), glutathione reductase (*gsr*), the family of glutathione peroxidases (*gpx*), the genes for the cytoplasmic (α, μ, ω, π, θ) glutathione *S*-transferases (*gst*), the microsomal and mitochondrial glutathione *S*-transferases (*mgst*, *gst13-13*), and the glutathione *S*-transferase *yc2* subunit. In addition, we examined one of the major, indirect mechanisms by which organophosphates produce oxidative stress, namely, their actions on the function and expression of ionotropic glutamate receptors (Damodaran et al. 2006; Gupta 2004), which mediate excitotoxic cell death in the developing brain, including that evoked by hypoxia (Choi and Rothman 1990). We assessed expression of the AMPA receptor family (*gria*), δ-subunits (*grid*), kainate receptors (*grik*), NMDA (*N*-methyl D-aspartate) receptors (*grin*), and their associated glutamate-binding protein (*grina*). By way of contrast, we also assessed the metabotropic glutamate receptors (*grm*), which are not involved in excitotoxicity. For reference, Table 1 lists all genes tested, with their full names and GenBank accession numbers (National Center for Biotechnology Information 2008).

In addition to comparing the effects of chlorpyrifos with those of diazinon, we evaluated two developmental neurotoxicants from different classes: dieldrin, an organochlorine pesticide, and divalent nickel (Ni^2+^). Both of these represent significant environmental concerns because like the organophosphates, they appear on the registry of Superfund chemicals (National Library of Medicine 2006). Although dieldrin acts primarily by blocking GABA_A_ receptors (Brannen et al. 1998; Liu et al. 1998), it also elicits oxidative stress akin to that of the organophosphates (Kitazawa et al. 2001, 2003; Slotkin et al. 2007b) and produces fetal brain damage (Uzoukwu and Sleight 1972). Ni^2+^ is found in the fetal brain in concentrations up to 2 μg/g, similar to lead (Casey and Robinson 1978), and, like lead, interferes with the gating of calcium during neurodifferentiation (Benters et al. 1996; Nikodijevic and Guroff 1992). In contrast to the other agents, Ni^2+^ does not elicit oxidative stress in developing neuronal cells (Slotkin et al. 2007b), and in fact, it shifts cellular redox status toward reduction, likely because it can donate electrons to form higher valence states (Slotkin et al. 2007b).

Because we wanted to compare the transcriptional responses inherent to each compound, we needed to avoid the confounds of pharmaco kinetic differences or effects on the maternal–fetal unit. Accordingly, we studied PC12 cells, a widely accepted *in vitro* model for neuronal development (Teng and Greene 1994) that has already been validated to reproduce the mechanisms and outcomes found after *in vivo* exposures of developing rats to organophosphates (Bagchi et al. 1995, 1996; Crumpton et al. 2000a, 2000b; Das and Barone 1999; Flaskos et al. 1994; Jameson et al. 2006, 2007; Li and Casida 1998; Nagata et al. 1997; Qiao et al. 2001, 2005; Slotkin et al. 2007a, 2007b, 2008d, 2008e; Song et al. 1998; Tuler et al. 1989). When nerve growth factor (NGF) is added to the culture medium, PC12 cells begin to differentiate, forming neuritic projections and acquiring electrical excitability and neuronal phenotypes (Fujita et al. 1989; Song et al. 1998; Teng and Greene 1994). The effects on neurodifferentiation in PC12 cells have been characterized previously for each of the four agents studied here (Qiao et al. 2001; Slotkin et al. 2007b; Slotkin and Seidler 2008, 2009), providing the necessary end points with which to interpret transcriptional responses. Finally, we compared effects in the undifferentiated and differentiating states for chlorpyrifos, as well as evaluating temporal responses for chlorpyrifos, diazinon, dieldrin, and Ni^2+^, so as to explore the role of critical developmental periods for vulnerability to oxidative stress and excitotoxicity.

## Materials and Methods

### Cell cultures

Because of the clonal instability of the PC12 cell line (Fujita et al. 1989), we performed the experiments on cells that had undergone fewer than five passages. As described previously (Qiao et al. 2003; Song et al. 1998), we seeded PC12 cells (American Type Culture Collection, 1721-CRL; obtained from the Duke Comprehensive Cancer Center (Durham, NC) onto poly-D-lysine–coated plates in RPMI-1640 medium (Invitrogen, Carlsbad, CA) supplemented with 10% inactivated horse serum (Sigma Chemical Co., St. Louis, MO), 5% inactivated fetal bovine serum (Sigma), and 50 μg/ mL penicillin streptomycin (Invitrogen). Incubations were carried out with 7.5% CO_2_ at 37°C, standard conditions for PC12 cells. To initiate neurodifferentiation (Jameson et al. 2006; Slotkin et al. 2007b; Teng and Greene 1994) 24 hr after seeding, the medium was changed to include 50 ng/mL of 2.5S murine NGF (Invitrogen). Along with the NGF, we added 30 μM of the test agent: chlorpyrifos (Chem Service, West Chester, PA), diazinon (Chem Service), dieldrin (Chem Service), or NiCl_2_ (Sigma). We chose this concentration from earlier studies that demonstrated adverse effects on differentiation of PC12 cells without outright cytotoxicity (Jameson et al. 2007; Qiao et al. 2001; Slotkin et al. 2007b, 2008d). Because of the limited water solubility of the three insecticides, we dissolved these agents in DMSO (final concentration, 0.1%), which was also added to the control cultures and to cultures containing NiCl_2_; this concentration of DMSO has no effect on PC12 cell growth or differentiation (Qiao et al. 2001, 2003; Song et al. 1998). We examined cultures 24 and 72 hr after commencing exposure, with five to eight independent cultures evaluated for each treatment at each time point. We used two time points so we could evaluate changes in gene expression regardless of whether the mRNA for a given gene has a rapid turnover (and hence can rise rapidly) or a slower turnover that would require a longer period to show corresponding increases or decreases. For chlorpyrifos, we evaluated the effects both on undifferentiated cells and during NGF-induced differentiation, whereas for the other agents, we studied only the effects during differentiation.

### Microarray determinations

In the present study, we performed mRNA isolation, preparation of cDNA, conversion to cRNA incorporating cyanine-3 (reference RNA) or cyanine-5 (sample RNA), verification of RNA purity and quality, hybridization to the micro-arrays, washing, and scanning as described previously (Slotkin et al. 2007c, 2008d; Slotkin and Seidler 2007). The mRNA used for the reference standard was created by pooling aliquots from each of the samples in the study. Similarly, array normalizations and error detection were carried out using procedures described previously (Slotkin et al. 2007c, 2008d; Slotkin and Seidler 2007). We used Agilent Whole Rat Genome Arrays (Agilent Technologies, Palo Alto, CA), type G4131A for the studies of chlorpyrifos in undifferentiated and differentiating cells, and type G4131F for the studies of diazinon, dieldrin, and Ni^2+^ in differentiating cells. The two chips contain exactly the same sequences, but the latter has a lower detection threshold; however, all the genes reported here passed the quality control filters with both arrays.

For many of the genes, the arrays contain multiple probes for the same gene and/or replicates of the same probe in different locations on the chip, and we used these to verify the reliability of values and the validity of the measures on the chip. To avoid artificially inflating the number of positive findings, we limited each gene to a single set of values, selecting those obtained for the probe showing the smallest intragroup variance. We used the other values for that gene only to corroborate direction and magnitude of change. We also validated the readings on the arrays with duplicate arrays for selected samples (Slotkin et al. 2007c; Slotkin and Seidler 2007).

### Statistical procedures

Because of the requirement to normalize the data across arrays and within each gene, the absolute values for a given gene are meaningless, so only the relative differences between treatments can be compared. Accordingly, we present results as means and SEs of the percent change from control values to allow for visual comparison of the effects across families of genes. However, statistical comparisons were based on the actual ratios (log-transformed, because the data are ratios) rather than the percent change.

Our design involved multiple planned comparisons of four agents at two time points, as well as the effects of one agent (chlorpyrifos) in undifferentiated versus differentiating states. It was therefore important to consider the false-positive rate and to protect against the increased probability of type 1 errors engendered by repeated testing of the same database. Accordingly, before looking at effects on individual genes, we performed a global analysis of variance (ANOVA) incorporating all the variables in a single comparison: treatment, time, and all genes. We then carried out lower-order ANOVAs on subdivisions of the data set as permitted by the interactions of treatment with the other variables. We evaluated differences for individual treatments for a specified gene at a single time point with Fisher’s protected least significant difference. However, for a given gene that showed no interaction of treatment with other variables (time, differentiation state), we report only the main treatment effect without subtesting effects at a single time point. We considered treatment effects significant at *p* < 0.05 (two-tailed, because we were interested in both increases and decreases in gene expression). Finally, concordance of patterns of effects between different agents was evaluated by linear regression analysis.

In addition to these parametric tests of the direction and magnitude of changes in gene expression, we evaluated the incidence of significant differences compared with the predicted false-positive rate, using Fisher’s exact test, applying a one-tailed criterion of *p* < 0.05, because only an increase above the false-positive rate would be predicted; at the criterion of *p* < 0.05, 1 gene of every 20 tested can be expected to show a difference at random. Finding a significant decrease in the incidence of detected differences relative to the false-positive rate would be biologically implausible and statistically meaningless.

## Results

We compared the effects on undifferentiated versus differentiating cells with only one agent (chlorpyrifos), so we performed two sets of global statistical tests. For chlorpyrifos, the multivariate ANOVA incorporated the factors of treatment, differentiation state, time, and gene and identified interactions of treatment × gene (*p* < 0.0001), treatment × differentiation state × gene (*p* < 0.0001), and treatment × time × state × gene (*p* < 0.03). Because of the strong interaction with differentiation state, we then subdivided the results to isolate the effects on undifferentiated and differentiating cells and identified significant effects in both states: undifferentiated, treatment × gene (*p* < 0.0001); differentiating, treatment × time (*p* < 0.04), treatment × gene (*p* < 0.0001), and treatment × time × gene (*p* < 0.05). Chlorpyrifos exposure evoked significant changes in the expression of 40 of the total of 59 genes, compared with an expected false-positive rate of only 3 genes (*p* < 10^−14^), and the same was true for the separate analyses of undifferentiated cells (24 of 59 genes, *p* < 10^−7^) and differentiating cells (34 of 59 genes, *p* < 10^−9^); in addition, the incidence of changes in differentiating cells was significantly greater than in the undifferentiated state (*p* < 0.05). In light of the significant ANOVA interaction terms, we separated data according to pathway groups and then performed lower-order tests for each gene.

For the study of diazinon, dieldrin, and Ni^2+^ conducted in differentiating cells, global ANOVA (factors of treatment, gene, time) identified a main effect of treatment (*p* < 0.0001) and interactions of treatment × time (*p* < 0.02), treatment × gene (*p* < 0.0001), and treatment × time × gene (*p* < 0.0001). Of the 59 total genes, we found significant differences for 44 (*p* < 10^−15^ vs. the predicted false-positive rate). This was also true for each agent considered individually: diazinon, 32 genes, *p* < 10^−10^; dieldrin, 26 genes, *p* < 10^−8^; Ni^2+^, 27 genes, *p* < 10^−8^. In light of the interactions of treatment with the other variables, we divided the data into the separate treatments for presentation, grouping the genes by pathway and evaluating the effects on each gene.

### Effects of chlorpyrifos in undifferentiated cells

**Figure 1:**
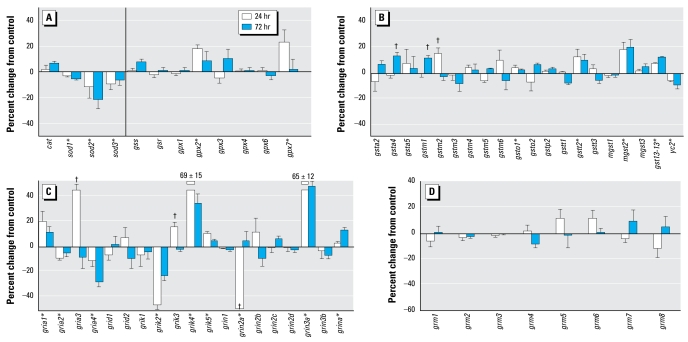
Effects of 30 μM chlorpyrifos exposure in undifferentiated PC12 cells. (*A*) Antioxidant genes. (*B*) Genes encoding the glutathione *S*-transferases. (*C*) Genes encoding the ionotropic glutamate receptors. (*D*) Genes encoding the metabotropic glutamate receptors. The vertical line in (*A*) separates genes encoding catalase and the superoxide dismutase isoforms from those involved in glutathione synthesis and redox status. *Significant main treatment effect. ^†^Treatment × time interaction and times for which treatment effects were present. Multivariate ANOVA (treatment, gene, time) indicates interactions of treatment × gene (*p* < 0.0001) and treatment × gene × time (*p*< 0.01).

Exposure of undifferentiated PC12 cells to chlorpyrifos for 24 or 72 hr had significant but modest effects on genes mediating anti-oxidant responses and glutathione metabolism. Although *cat* expression was unaffected, all three *sod* subtypes showed small but significant down-regulation (Figure 1A). Neither *gss* nor *gsr* was affected, but two out of the six *gpx* genes showed significant up-regulation. The genes encoding the glutathione *S*-transferases likewise showed statistically significant changes in response to chlorpyrifos exposure, but the magnitude of effect did not exceed 20% (Figure 1B). In general, the main effect was up-regulation (*gsta4*, *gstm1*, *gstm2*, *gsto1*, *gstt2*, *mgst2*, *gst13-13*), with the exception of *yc2*, which showed a decrement.

In contrast, we identified widespread and robust effects of chlorpyrifos on the gene family for ionotropic glutamate receptors (Figure 1C). We found consistent increases for *gria1*, *grik4*, *grik5*, *grin3a*, and *grina* and persistent decreases for *gria2*, *gria4*, and *grik2*. In addition, some genes showed transient effects, with increases (*gria3*, *grik3*) or decreases (*grin2a*) after 24 hr of exposure that waned by 72 hr. We did not see these effects for the metabotropic glutamate receptors, none of which showed significant effects of chlorpyrifos in undifferentiated cells (Figure 1D).

### Antioxidant genes in differentiating cells

**Figure 2:**
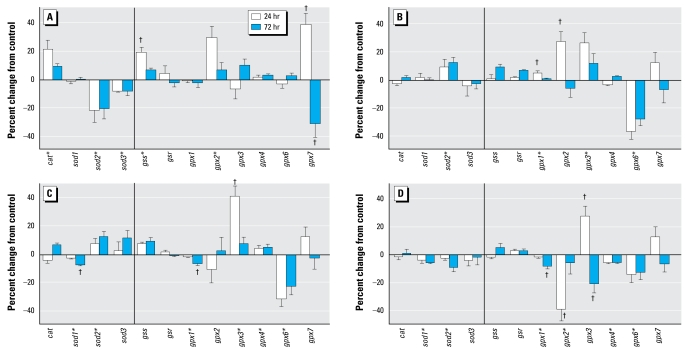
Effects of 30 μM chlorpyrifos (*A*), diazinon (*B*), dieldrin (*C*), and Ni^2+^ (*D*) exposure on expression of antioxidant genes in differentiating PC12 cells. The vertical lines separate genes encoding catalase and the superoxide dismutase isoforms from those involved in glutathione synthesis and redox status. *Significant main treatment effect. ^†^Treatment × time interaction and times for which treatment effects were present. Multivariate ANOVA (all treatments, all genes, time) indicates a significant main effect of treatment (*p* < 0.02) and interactions of treatment × gene ( *p* < 0.0001) and treatment × gene × time (*p* < 0.006).

Compared with the effects on undifferentiated PC12 cells, chlorpyrifos exposure during differentiation elicited a greater overall response for the genes involved in antioxidant activity (Figure 2A). Across all these genes, the absolute magnitude of effect was doubled (net 12% change in differentiating cells vs. 6% in undifferentiated cells, *p* < 0.02). Chlorpyrifos elicited significant up-regulation of *cat* and transient up-regulation of *gss*, effects that were not seen in the undifferentiated state, as well as eliciting much larger initial increases in *gpx2* and *gpx7*; for the latter, we found a significant subsequent rebound suppression at 72 hr. Two additional changes resembled those seen in the undifferentiated cells, namely, down-regulation of *sod2* and *sod3*.

The response to diazinon in differentiating PC12 cells was distinctly different from that evoked by chlorpyrifos (Figure 2B). Diazinon failed to alter *cat*, *gss*, or *gpx7* expression; enhanced *sod2* instead of suppressing it; and evoked additional changes not seen with chlorpyrifos, namely, increases in *gpx1* and *gpx3* and decreases in *gpx6*. The only point of clear overlap between the two agents was for *gpx2*, which showed the same transient elevation for diazinon and for chlorpyrifos.

Dieldrin evoked some of the same changes as did diazinon, including up-regulation of *sod2* and *gpx3* and down-regulation of *gpx6* (Figure 2C). It differed in that dieldrin reduced *sod1*, *gpx1*, and *gpx4*, leaving *gpx2* unaffected. Exposure to Ni^2+^ produced a different response pattern, with small but significant reductions in *sod1*, *sod2*, *gpx1*, and *gpx6* and a larger transient decrease in *gpx2* (Figure 2D). The only major point of consonance was for *gpx3*, which showed the same type of transient increase as seen with diazinon and dieldrin, except that with Ni^2+^, it was followed by a decrease at 72 hr.

### Glutathione S-transferases in differentiating cells

**Figure 3:**
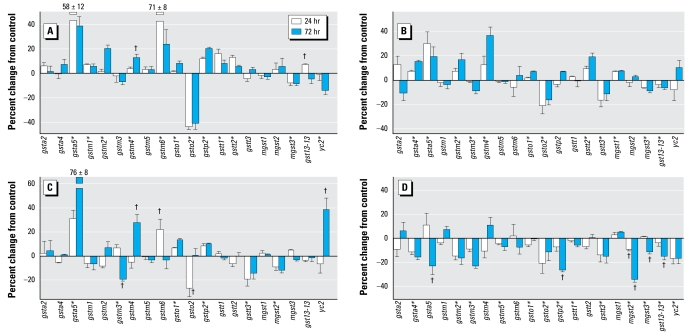
Effects of 30 μM chlorpyrifos (*A*), diazinon (*B*), dieldrin (*C*), and Ni^2+^ (*D*) exposure on expression of genes encoding the glutathione *S*-transferases in differentiating PC12 cells. *Significant main treatment effect. ^†^Treatment × time interaction and times for which treatment effects were present. Multivariate ANOVA (all treatments, all genes, time) indicates a significant main effect of treatment (*p* < 0.0001) and interactions of treatment × time ( *p* < 0.001), treatment × gene (*p*< 0.0001), and treatment × gene × time (*p* < 0.0001).

Again, the genes encoding the various glutathione S-transferases showed much greater responses to chlorpyrifos in differentiating PC12 cells (Figure 3A) than in undifferentiated cells, with double the absolute magnitude of change (net 12% in differentiating cells vs. 6% in undifferentiated cells, *p* < 0.03). Indeed, we found significant changes in expression for 13 of the 18 genes in this group, with increases (10 genes) predominating over decreases (3 genes). The largest changes were seen for *gsta5* (increase), *gstm6* (increase), and *gsto2* (decrease). We found smaller increases for *gstm1*, *gstm2*, *gstm4*, *gsto1*, *gstp2*, *gstt1*, *gstt2*, and *gst13-13*; minor but significant decreases were confined to *mgst3* and *yc2*.

For this set of genes, diazinon exposure altered expression in a pattern somewhat similar to that of chlorpyrifos, albeit with much smaller overall effects on the genes that showed the biggest changes with chlorpyrifos (Figure 3B). Like chlorpyrifos, diazinon evoked up-regulation of *gsta5*, *gstm2*, *gstm4*, *gsto1*, and *gstt2* and down-regulation of *gsto2* and *mgst3*. In addition, diazinon enhanced *gsta4* and suppressed *gstm3*, *gstt3*, and *gst13-13*, effects that were not seen with chlorpyrifos, while failing to affect many of the genes that were significantly altered by chlorpyrifos (*gstm1*, *gstm6*, *gstp2*, *gstt1*, *yc2*). With dieldrin exposure (Figure 3C), we again saw features shared by chlorpyrifos and/or diazinon, namely, robust up-regulation of *gsta5*; lesser stimulation of *gstm4*, *gstm6*, *gsto1*, and *gstp2*; and down-regulation of *gstm3*, *gsto2*, and *gstt3*. We also found features unique to dieldrin: a decrease in *mgst2* and an increase in *yc2* expression. Although exposure to Ni^2+^ (Figure 3D) produced some changes in gene expression resembling those of the other three agents (e.g., decreased *gsto2* expression), the overall response pattern was distinctly different, with inhibition predominating over stimulation (main treatment effect, *p* < 0.0001). We found significant decreases for all but 6 of the 20 genes, and no individual gene showed a significant increase.

### Glutamate receptors in differentiating cells

**Figure 4:**
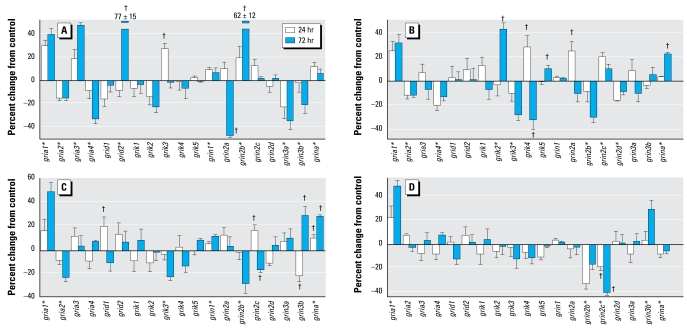
Effects of 30 μM chlorpyrifos (*A*), diazinon (*B*), dieldrin (*C*), and Ni^2+^ (*D*) exposure on expression of genes encoding the ionotropic glutamate receptors in differentiating PC12 cells. *Significant main treatment effect. ^†^Treatment × time interaction and times for which treatment effects were present. Multivariate ANOVA (all treatments, all genes, time) indicates interactions of treatment × time (*p* < 0.01), treatment × gene ( *p* < 0.0001), and treatment × gene × time (*p* < 0.0003).

Unlike the situation with the genes involved in oxidative stress, chlorpyrifos exposure in differentiating cells showed approximately the same net absolute response for glutamate receptor genes as in undifferentiated cells (16% change for differentiating cells vs. 12% change for undifferentiated cells; not significant); this was also true even if the comparison was restricted to ionotropic glutamate receptors (18% vs. 16%, respectively), the sub group that participates in organophosphate-induced neuro toxicity (Chebabo et al. 1999; Damodaran et al. 2006; Dekundy et al. 2007; Gupta 2004). However, we found significant differences in the response of specific genes in the differentiating versus undifferentiated states (treatment × state × gene, *p* < 0.004) and in the time course of effect (treatment × state × gene × time, *p* < 0.006). Just as in undifferentiated cells, chlorpyrifos exposure during differentiation elicited significant up-regulation of *gria1*, *gria3*, *grik3*, and *grina* and down-regulation of *gria2*, *gria4*, and *grin2a* (Figure 4A). However, we also found key differences: increases in *grid2*, *grin1*, and *grin2b* and decreases in *grin3b* restricted to differentiating cells (Figure 4A); increases in *grik4* and *grik5*, and decreases in *grik2* restricted to undifferentiated cells (Figure 1C); increases in *grin3a* in undifferentiated cells (Figure 1C) but decreases for the same gene in differentiating cells (Figure 4A); and differences between undifferentiated (Figure 1C) and differentiating cells (Figure 4A) in the time course of effect for *gria3* and *grin2a*.

Like chlorpyrifos, diazinon exposure altered the expression for most of the genes encoding ionotropic glutamate receptors (Figure 4B). However, the patterns of effects showed major disparities between the two organophosphates, with similar changes for only four genes (*gria1*, *gria2*, *gria4*, *grina*) and dissimilar effects for 11 genes (*gria3*, *grid2*, *grik2*, *grik3*, *grik4*, *grik5*, *grin2a*, *grin2b*, *grin2d*, *grin3a*, *grin3b*), even to the extent of changes in the opposite direction. Dieldrin elicited more modest changes, but in general, the pattern was closer to that of diazinon, sharing similar changes for *gria1*, *gria2*, *grik3*, *grin2b*, *grin2c*, and *grina*, as well as a lack of effect on *gria3*, *grid2*, *grik1*, and *grin3a* (Figure 4C); the two differed for *gria4*, *grid1*, *grik2*, *grik4*, and *grin2a*. Although exposure to Ni^2+^ elicited the same increase in *gria1* seen with the other three agents, in general it affected a much smaller repertoire of genes, with only five significant changes among the total of 19 (Figure 4D). Like diazinon or dieldrin, Ni^2+^ reduced the expression of *grin2b*, an effect opposite that of chlorpyrifos, but Ni^2+^ also affected *grin2c* (decrease), *grina* (decrease), and *grin3b* (increase) in a manner distinct from one or more of the other agents.

**Figure 5:**
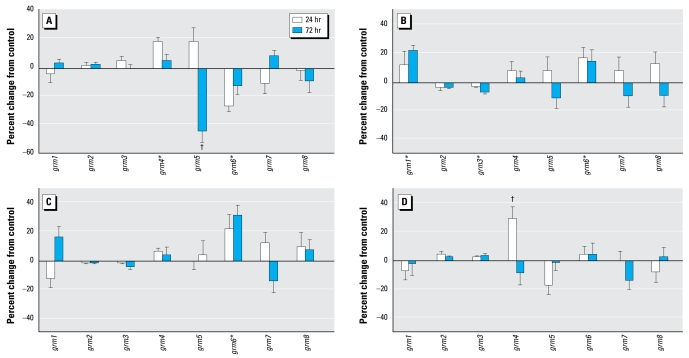
Effects of 30 μM chlorpyrifos (*A*), diazinon (*B*), dieldrin (*C*), and Ni^2+^ (*D*) exposure on expression of genes encoding the metabotropic glutamate receptors in differentiating PC12 cells. *Significant main treatment effect. ^†^Treatment × time interaction and times for which treatment effects were present. Multivariate ANOVA (all treatments, all genes, time) indicates interactions of treatment × time (*p* < 0.05) and treatment × gene ( *p* < 0.02).

Although chlorpyrifos exposure in undifferentiated PC12 cells did not alter the expression of genes encoding the metabotropic glutamate receptors, differentiating cells were more sensitive, displaying changes for three of the eight subtypes (Figure 5A): increased *grm4* and decreased *grm5* and *grm6*. The effects of diazinon were totally different, with increases in *grm1* and *grm6* and a small but significant decrease in *grm3* (Figure 5B). Dieldrin produced even less of an effect, restricted to an increase in *grm6* (Figure 5C). Likewise, Ni^2+^ evoked only a transient change in *grm4* expression (Figure 5D). Taken across all four agents, the incidence of changes in metabotropic glutamate receptor genes (25%) was much lower than for ionotropic receptors (50%, *p*< 0.01 by Fisher’s exact test).

## Discussion

In earlier work with the PC12 cell model, we showed that lipid peroxidation evoked by chlorpyrifos was enhanced by coexposure to NGF (Qiao et al. 2005), consistent with increased sensitivity to oxidative stress during neuro differentiation. We confirmed this by studies conducted *in vivo* with chlorpyrifos administered to neonatal rats, which likewise evoked greater lipid peroxidation in vulnerable brain regions during peak periods of axonogenesis and synapto genesis (Slotkin et al. 2005). In the present study, we found much larger and widespread transcriptional changes elicited by chlorpyrifos exposure in differentiating PC12 cells compared with undifferentiated cells, for genes involved in the oxidative stress response (*cat*, *sod*, *gss*, *gsr*, *gpx*, *gst*, *mgst*, *yc2*; including all subtypes); the correspondence of the changes in gene expression with the functional end point of lipid peroxidation thus serves as a validation of this approach. Further, genes encoding the glutamate receptors did not show a corresponding net increase in chlorpyrifos effects during differentiation, demonstrating the specificity for those elements directly involved in mustering anti oxidant defenses; we found selective differences for individual receptor genes that depended on differentiation state, but the overall magnitude of effect was no greater in differentiating cells compared with undifferentiated cells, unlike the situation for anti-oxidant genes. Indeed, for undifferentiated cells, the effect of chlorpyrifos on ionotropic glutamate receptor genes was far more robust than for the genes involved in oxidative stress; further, this was not true for metabotropic glutamate receptors, which are not involved in excitotoxicity. Our results thus suggest a greater role for excitotoxicity than for oxidative stress in the undifferentiated state (i.e., earlier stages of neurodevelopment) but an increasing role for oxidative stress as cells undergo differentiation (later stages of neurodevelopment). The comparative effects of chlorpyrifos in the two states thus reinforce the idea that both oxidative stress and excitotoxicity are likely contributors that define the critical windows in which specific neuronal populations in the developing brain are most vulnerable to different aspects of chlorpyrifos neurotoxicity (Garcia et al. 2005; Slotkin 1999, 2004, 2005).

In differentiating cells, both chlorpyrifos and diazinon produced widespread changes in the expression of genes involved in oxidative stress. Although we found a significant correlation between the effects on these genes for the two organophosphates (Table 2), the relatively modest correlation coefficient (*r* = 0.40) points to substantial differences, as well. In fact, the effects on the primary anti oxidant genes—*cat* and the *sod* subtypes—were totally dissimilar, as evidenced by a strong negative correlation between chlorpyrifos and diazinon (Table 2); this reflected not only an opposite direction of change for *sod2* but also a substantially greater overall effect of chlorpyrifos across this set of genes (average change 11% for chlor pyrifos vs. 4% for diazinon, *p* < 0.02). These results point to the likelihood that chlorpyrifos either produces a greater degree of oxidative stress or, through its other mechanisms of action, exacerbates the net effect of oxidative stress. In contrast, our results for the glutathione-related genes suggest a similar outcome from the two agents, evidenced by a significant positive correlation (Table 2); the larger number of glutathione-related genes also accounts for the overall concordance between chlorpyrifos and diazinon across all the oxidative-stress–related genes and points to why examination of subdivisions is important to delineate different mechanisms and potential outcomes. Indeed, despite the positive correlation for the glutathione-related genes, individual components in that set displayed markedly different effects of the two agents (e.g., *gpx6* and *gstm6*).

The much more widespread and robust effects of chlorpyrifos on expression of ion-otropic glutamate receptors compared with metabotropic receptors in differentiating PC12 cells reflects the specific involvement of these receptors in the excitotoxicity associated with organophosphate-induced neuronal injury (Damodaran et al. 2006; Gupta 2004) and are similar to results reported previously for effects on the developing rat brain after neonatal chlorpyrifos exposure (Slotkin and Seidler 2007). Here, although diazinon likewise produced widespread effects on ionotropic glutamate receptors, the overall pattern was totally distinct from that of chlorpyrifos, evidenced by a lack of concordance whether considering each receptor class separately or together (Table 2). Again, this is consistent with major differences between the two agents observed for expression of the same receptor genes after *in vivo* exposures (Slotkin and Seidler 2007). One likely reason is that chlor pyrifos may interact directly with ion channel receptors that gate calcium entry (Katz et al. 1997; Smulders et al. 2004). Because this mechanism obviously reflects actions unrelated to the shared property of cholinesterase inhibition, the structural differences between chlorpyrifos and diazinon could clearly contribute to dissimilar effects on ionotropic glutamate receptor function, leading to disparities in receptor expression.

Perhaps the most surprising result is the close similarity between the outcomes of exposure to dieldrin and those of diazinon. We found high concordance between these two agents across the oxidative-stress–related genes as well as the glutamate receptor genes, resulting in a substantially higher correlation across all genes than for chlorpyrifos and diazinon, the two organophosphates (Table 2). This was also reflected in the lesser correlations between chlorpyrifos and dieldrin, which were just as robust as those between chlorpyrifos and diazinon (Table 2). In our earlier work with PC12 cells, we similarly found convergent outcomes for diazinon and dieldrin on lipid peroxidation and on differentiation end points, including indices of neurite formation and neurotransmitter phenotype (Slotkin et al. 2007b; Slotkin and Seidler 2008, 2009). Our present findings can thus guide future studies of *in vivo* dieldrin exposure to confirm the prediction that this agent will produce developmental neurotoxicity akin to that of diazinon.

Ni^2+^ does not evoke oxidative stress (Slotkin et al. 2007b), and accordingly, this agent elicited changes in gene expression that were distinct from those of the organophosphates. We found no significant concordance in the outcomes for chlorpyrifos compared with Ni^2+^ and only a weak overall correlation for diazinon and Ni^2+^ that did not achieve significance for any subset of genes (Table 2). The difference between Ni^2+^ and the pesticides is best illustrated by comparing effects on the glutathione *S*-transferases, where the metal produced widespread down-regulation compared with the predominance of up-regulation for three pesticides. Further, the fact that all four agents (including Ni^2+^) down-regulated *gsto2* indicates that this particular transcriptional change is probably not involved in the response to oxidative stress per se. We found a significant overall concordance for dieldrin and Ni^2+^, primarily reflecting their shared actions on glutamate receptors. Interestingly, although we found concordance between dieldrin and Ni^2+^ and between diazinon and dieldrin for the ionotropic glutamate receptors, the correlation was much poorer between dieldrin and Ni^2+^; this points out that the concordance patterns between any two agents can involve sets of genes different from those generating the concordance between one of those agents and a third compound. In any case, the unexpected similarities between apparently unrelated neurotoxicants in their effects on ionotropic glutamate receptors points to future experiments on the potential for convergent underlying mechanisms and outcomes involving excitotoxicity.

The limitations and advantages of the combination of *in vitro* model and planned comparison approaches to micro array data have been detailed in earlier work (Slotkin et al. 2007c, 2008d, 2008e; Slotkin and Seidler 2007, 2008) but are worth repeating in relation to the present study. We used cells from a transformed cell line, which, unlike primary neurons in culture, maintain their ability to divide, an important consideration when, as here, the neurotoxicants target the cell cycle as part of their injury pattern. Nevertheless, transformed cells are inherently less sensitive to toxicant injury than are developing neurons *in vivo*. Further, cell culture treatments involve much shorter durations than with environmental exposures extending throughout brain development. We considered both of these factors in our selection of the 30 μM test concentrations. In the case of the organophosphates, this is approximately an order of magnitude higher than the levels in newborn babies after nonsymptomatic environmental exposures in agricultural communities (Ostrea et al. 2002); however, the cultures contain high concentrations of serum proteins, so < 10% of the nominal concentration is actually available to diffuse into the cells (Qiao et al. 2001). The most important proof of relevance, however, is that for chlorpyrifos and diazinon, parallel outcomes have been identified between *in vivo* exposures and the PC12 model (Bagchi et al. 1995, 1996; Crumpton et al. 2000a, 2000b; Das and Barone 1999; Flaskos et al. 1994; Jameson et al. 2006, 2007; Li and Casida 1998; Nagata et al. 1997; Qiao et al. 2001, 2005; Slotkin et al. 2007a, 2007b, 2008d, 2008e; Song et al. 1998; Tuler et al. 1989), thus providing validation of the *in vitro* approach. The second factor in these studies is our use of planned comparisons, a distinctly different approach from the global examination of the tens of thousands of genes present on the microarrays. Planned comparisons are based on testing a specific hypothesis that centers around a defined set of gene families and rests on known, validated outcomes from prior work, in this case involving both *in vivo* and *in vitro* demonstrations of oxidative stress from the organophosphates. With examination of the entire genome, verification via reverse transcriptase polymerase chain reaction and other techniques is required because the enormous number of comparisons generates numerous false-positive findings (e.g., the > 2,000 genes that would be false positives if we had considered all 42,000 probes on the array). For our study, we compared only a handful of genes that would generate only three false positives, and we found alterations in most of these genes; for interpretation, we relied on multiple gene changes in a given pathway, as well as effects that repeated across different treatments and/or different time points, rather than changes in any one gene. The odds of all those genes being false positives are astronomically small. However, even for individual genes, a given array used multiple probes and multiple spots (see “Materials and Methods”), so the changes cannot be “chance.” Unlike many array studies, where a single mRNA set derived from multiple samples might be evaluated, we evaluated up to eight separate samples for each treatment condition, so again, it is inconceivable statistically that we could produce these outcomes by accident. Indeed, one of the key points of this study is to demonstrate that a planned comparison approach may provide a superior strategy for the use of microarray data, provided that the relevant target pathways are known in advance.

In conclusion, our findings reinforce the growing body of evidence that the various organophosphates differ in their underlying mechanisms of developmental neurotoxicity, over and above their shared property as cholinesterase inhibitors, culminating in distinct outcomes at the levels of synaptic function (Jameson et al. 2007; Qiao et al. 2001; Roegge et al. 2008; Slotkin et al. 2006a, 2006b, 2007b, 2007c, 2008a, 2008b, 2008c, 2008d; Slotkin and Seidler 2007; Timofeeva et al. 2008a, 2008b). As shown here, these mechanisms are likely to include selective effects on oxidative stress and excitotoxicity, as well as enhanced vulnerability to oxidative stress during a critical period centered around differentiation into neurotransmitter phenotypes and the development of neuritic projections. At the same time, the concordance of overall effects between diazinon and dieldrin, and for effects of dieldrin and Ni^2+^ on glutamate receptors, indicate that agents from apparently unrelated classes of toxicants can nonetheless converge on common final outcomes, despite differences in underlying targets or originating mechanisms. Finally, the results obtained here illustrate how a combined use of cell culture systems and microarrays can guide future studies toward specific end points that can distinguish similarities and disparities in the effects of diverse developmental neurotoxicants.

## Figures and Tables

**Table 1:** Gene names and Genbank accession numbers.

Gene symbol	Gene name	GenBank accession no.
*cat*	catalase	NM_012520
*gpx1*	glutathione peroxidase 1	NM_030826
*gpx2*	glutathione peroxidase 2	BQ196649
*gpx3*	glutathione peroxidase 3	AI172411
*gpx4*	glutathione peroxidase 4	NM_017165
*gpx6*	glutathione peroxidase 6	NM_147165
*gria1*	glutamate receptor, ionotropic AMPA 1	NM_031608
*gria2*	glutamate receptor, ionotropic AMPA 2	NM_017261
*gria3*	glutamate receptor, ionotropic AMPA 3	NM_032990
*gria4*	glutamate receptor, ionotropic AMPA 4	NM_017263
*grid2*	glutamate receptor, ionotropic δ1	NM_024379
*grik1*	glutamate receptor, ionotropic kainate 1	AI111480
*grik2*	glutamate receptor, ionotropic kainate 2	NM_019309
*grik3*	glutamate receptor, ionotropic kainate 3	NM_181373
*grik4*	glutamate receptor, ionotropic kainate 4	NM_012572
*grik5*	glutamate receptor, ionotropic kainate 5	NM_017262
*grin1*	glutamate receptor, ionotropic *N*-methyl-D-aspartate 1	NM_017010
*grin2a*	glutamate receptor, ionotropic *N*-methyl-D-aspartate 2a	NM_012573
*grin2b*	glutamate receptor, ionotropic *N*-methyl-D-aspartate 2b	NM_012574
*grin2d*	glutamate receptor, ionotropic *N*-methyl-D-aspartate 2d	NM_022797
*grin3a*	glutamate receptor, ionotropic *N*-methyl-D-aspartate 3a	AF061945
*grin3b*	glutamate receptor, ionotropic *N*-methyl-D-aspartate 3b	NM_133308
*grina*	glutamate receptor, ionotropic *N*-methyl-D-aspartate-associated protein	NM_153308
*grm1*	glutamate receptor, metabotropic 1	NM_017011
*grm2*	glutamate receptor, metabotropic 2	XM_343470
*grm3*	glutamate receptor, metabotropic 3	M92076
*grm4*	glutamate receptor, metabotropic 4	NM_022666
*grm5*	glutamate receptor, metabotropic 5	NM_017012
*grm6*	glutamate receptor, metabotropic 6	NM_022920
*grm7*	glutamate receptor, metabotropic 7	NM_031040
*grm8*	glutamate receptor, metabotropic 8	NM_022202
*gsr*	glutathione reductase	NM_053906
*gss*	glutathione synthetase	NM_012962
*gst13-13*	glutathione *S*-transferase, mitochondrial	NM_181371
*gsta2*	glutathione *S*-transferase, α 2	BQ199390
*gsta4*	glutathione *S*-transferase, α 4	XM_217195
*gstm1*	glutathione *S*-transferase, μ1	NM_017014
*gstm2*	glutathione *S*-transferase, μ2	NM_177426
*gstm3*	glutathione *S*-transferase, μ3	NM_031154
*gstm4*	glutathione *S*-transferase, μ4	NM_020540
*gstm5*	glutathione *S*-transferase, μ5	NM_172038
*gstm6*	glutathione *S*-transferase, μ6	XM_215682
*gsto1*	glutathione *S*-transferase, ω1	NM_001007602
*gsto2*	glutathione *S*-transferase, ω2	NM_001012071
*gstp2*	glutathione *S*-transferase, π2	NM_138974
*gstt1*	glutathione *S*-transferase, θ1	NM_053293
*gstt2*	glutathione *S*-transferase, θ2	NM_012796
*gstt3*	glutathione *S*-transferase, θ3	XM_574740
*mgst1*	microsomal glutathione *S*-transferase 1	NM_134349
*mgst2*	microsomal glutathione *S*-transferase 2	XM_215562
*mgst3*	microsomal glutathione *S*-transferase 3	XM_213943
*sod1*	superoxide dismutase 1	NM_017050
*sod2*	superoxide dismutase 2	AI235842
*sod3*	superoxide dismutase 3	NM_012880
*yc2*	glutathione *S*-transferase yc2 subunit	NM_001009920

**Table 2:** Concordance between test agents.

	Chlorpyrifos vs. diazinon	Chlorpyrifos vs. dieldrin	Chlorpyrifos vs. Ni^2+^	Diazinon vs. dieldrin	Diazinon vs. Ni^2+^	Dieldrin vs Ni^2+^
All oxidative stress- and glutathione-related genes*a*	*r* = 0.40	*r* = 0.37	*r* = 0.11	*r* = 0.62	*r* = 0.20	*r* = 0.30
	*p* < 0.001	*p* < 0.003	NS	*p* < 0.0001	NS	*p* < 0.02

*cat*, *sod*	*r* = −0.66	*r* = −0.62	*r* = 0.53	*r* = 0.51	*r* = −0.52	*r* = −0.08
	*p* < 0.05	*p* < 0.05	NS	NS	NS	NS

All glutathione-related genes	*r* = 0.46	*r* = 0.43	*r* = 0.13	*r* = 0.63	*r* = 0.22	*r* = 0.31
	*p* < 0.0004	*p* < 0.002	NS	*p* < 0.0001	NS	*p* < 0.03

All glutamate receptor genes	*r* = 0.02	*r* = 0.02	*r* = 0.03	*r* = 0.58	*r* = 0.23	*r* = 0.56
	NS	NS	NS	*p* < 0.0001	NS	*p* < 0.0001

Ionotropic glutamate receptors	*r* = 0.05	*r* = 0.06	*r* = 0.02	*r* = 0.59	*r* = 0.25	*r* = 0.60
	NS	NS	NS	*p* < 0.0001	NS	*p* < 0.0001

Metabotropic glutamate receptors	*r* = 0.09	*r* = −0.37	*r* = −0.02	*r* = 0.60	*r* = 0.05	*r* = 0.31
	NS	NS	NS	*p* < 0.02	NS	NS

All genes	*r* = 0.20	*r* = 0.18	*r* = 0.04	*r* = 0.60	*r* = 0.21	*r* = 0.42
	*p* < 0.04	*p* < 0.05	NS	*p* < 0.0001	*p* < 0.03	*p* < 0.0001

NS, not significant.

a All genes in the *cat*, *sod*, *gss*, *gsr*, *gpx*, and *gst* families.
